# Advance Care Planning in German General Practice: A Longitudinal Qualitative Study on Patients' Expectations and Experiences

**DOI:** 10.1111/hex.70392

**Published:** 2025-08-17

**Authors:** Alexandra Schmidt, Klaus Weckbecker, Jürgen in der Schmitten, Kornelia Götze, Achim Mortsiefer

**Affiliations:** ^1^ Chair of General Practice II and Patient‐Centredness in Primary Care, Institute of General Practice and Primary Care (iamag), Faculty of Health Witten/Herdecke University Witten Germany; ^2^ Chair of General Practice I and Interprofessional Care, Institute of General Practice and Primary Care (iamag), Faculty of Health Witten/Herdecke University Witten Germany; ^3^ Institute of Family Medicine/General Practice, Medical Faculty University of Duisburg‐Essen Essen Germany; ^4^ Institute of General Practice, Center of Health and Society (chs), Medical Faculty, Heinrich Heine University Düsseldorf Germany

**Keywords:** ACP, advance care planning, advance directive, general practice, living will, patient autonomy, primary healthcare

## Abstract

**Background:**

Advance care planning (ACP) empowers individuals to make informed decisions about future medical care. While research has focused on patients with advanced chronic or terminal conditions, there remains a notable gap in understanding the perspectives and experiences of ambulatory individuals who proactively engage in ACP.

**Objective:**

To explore ambulatory patients' perspectives on and experiences with facilitated ACP provided by trained professionals in German general practices, focusing on the expectations, motivations and outcomes of the ACP process.

**Design:**

Longitudinal qualitative design using semi‐structured telephone interviews, conducted before, immediately after and 12 months after ACP facilitation. Interviews were analysed using thematic qualitative text analysis by Kuckartz.

**Setting and Participants:**

Interviews were conducted with eight German patients (six females and two males; mean age: 63.6 years; frailty score from 1 to 5) in two general practice settings.

**Results:**

Participants valued comprehensive advance directives (ADs) and structured ACP conversations in general practice. Key motivators for seeking ACP facilitation were maintaining autonomy and reducing family burden. Trustworthy discussions with professional facilitators, structured guidance and clear communication, particularly involving healthcare proxies, were essential for a satisfying ACP process. Despite expressing a prior interest in discussing care preferences, some participants had not yet initiated these conversations.

**Conclusions:**

ACP facilitation enables patients to articulate their values and healthcare preferences, supporting decision‐making and reducing uncertainty. Integrating structured ACP discussions into routine primary care could enhance patient autonomy and preparedness. Future research should explore strategies to optimise ACP implementation and assess its long‐term impact on patient outcomes.

**Patient or Public Contribution:**

Stakeholder and patient involvement is a crucial aspect of empowering research subjects and improving study feasibility and relevance. In this study, stakeholders such as experts in the field of ACP (one female GP and two male GPs), other GPs and patients were involved as an expert panel in a stepwise participatory approach. The process involved training in ACP facilitation for AS before conducting the study, expert panel meetings to identify research gaps and prioritise research topics, focus group discussions to conceptualise study design and data collection procedures, as well as the development of interview guidelines and data analysis. A patient advisory board (seven females and four males; age: 36–78 years with chronic illnesses) associated with the Institute of General Practice and Primary Care (iamag) was also involved in creating the interview guidelines (guidelines can be found in Appendix 2–4).

**Trial Registration:**

German Clinical Trial Register (DRKS) (ID: DRKS00027368; date of registration: April 2023; URL: https://www.drks.de/DRKS00027368).

**Abbreviations**
ACP: Advance care planning
AD/ADs: Advance directive/Advance directives
GP: General practitioner

## 1 Introduction

Advance care planning (ACP) is a structured process designed to help individuals engage in meaningful discussions and on that basis eventually document their medical treatment preferences in an advance directive (AD). ACP aims to ensure that patients' well‐informed preferences are respected in situations where they may no longer be able to provide informed consent, aligning medical care with patients' values [1, 2, 3, 4, 5, 6].

Recognising the need for better ACP implementation, the German Hospice and Palliative Care Act of 2015 introduced ACP facilitation for residents in nursing homes and care homes for individuals with disabilities [7, 8, 9], providing access to facilitated ACP conversations covered by statutory health insurance. However, ACP in institutional settings is often initiated too late, when patients are already unable to provide informed consent. Therefore, earlier engagement in ACP is crucial, particularly in ambulatory care settings where individuals may still have the capacity to reflect on their values and eventually make informed decisions [7]. Research suggests that general practitioners (GPs) and their teams are well‐positioned to facilitate ACP conversations due to their long‐term relationships with patients [8]. However, time constraints, insufficient training and systemic and emotional barriers frequently hinder the implementation of ACP in primary care [9, 10, 11, 12].

While ACP has been widely studied among patients with advanced chronic or terminal illnesses, there is a lack of research on its application for ambulatory individuals with early signs of frailty or less severe health conditions. Despite the recognised benefits of ACP, little is known about the experiences of ambulatory patients engaging in facilitated ACP within general practice settings.

This study seeks to address this gap by exploring the perspectives of patients participating in a structured ACP programme [13] facilitated by trained professionals in German general practices. By focusing on patients' experiences and motivations, as well as on barriers and facilitators of the process, the study aims to contribute to the understanding of how ACP can be effectively integrated into primary care by answering the following research questions:

*Expectations of patients before ACP facilitation*

1. Goals, wishes and motivations: What motivated patients to engage in the ACP process?
2. Preconceptions: What are the views and what is the understanding of patients in general practice regarding ACP and ADs before their ACP conversations?

*Experiences of patients after ACP facilitation*

3. Perceptions: What factors are perceived as important for a successful ACP process?
4. Outcomes: What benefits do patients perceive from their ACP experience in general practice?

## 2 Methods

We employed an exploratory prospective study design utilising semi‐structured interviews to gain a comprehensive understanding of patients' experiences with the ACP process. The study is reported in accordance with the COREQ guideline (Appendix 1) [14].

### 2.1 Providing ACP Facilitation in General Practice

The study was conducted in two general practices located in small towns in North Rhine‐Westphalia, where ACP facilitation is routinely offered independently of the study. The average cost for ACP facilitation (€180 per process) was reimbursed to study participants.

All ACP facilitators (two GPs: male, 55 and 56 years; one medical assistant: female, 46 years) had completed ACP facilitator training. This training adheres to the standards of the ACP Germany Society (www.acp-d.org), with detailed curriculum and qualification guidelines found in the practice book for ACP [15]. In accordance with consensus definitions and experience from multiple ACP programmes, leading ACP conversations requires specific attitudes and communication competencies. Therefore, the term ‘qualified’ in this paper implies that the professional leading the ACP conversation has successfully completed such a standardised comprehensive training programme.

The ACP process involved patients having two 30‐ to 60‐min conversations with a qualified facilitator. This facilitation helped individuals clarify treatment goals, explore personal attitudes towards life, severe illness and death, and identify specific preferences for three separate medical scenarios [13]. As ACP is explicitly viewed as a lifelong process, all patients are actively encouraged to review their AD at regular intervals (every 1–5 years if clinically stable) or in response to health changes [15].

## 3 Participants and Setting

Participants were selected using convenience sampling. Study participants were patients who had either actively sought or been invited to ACP facilitation in one of the two participating general practices. Patients over the age of 18, capable of providing consent and fluent in German were included. To capture diverse perspectives, we aimed at purposeful sampling by including male and female patients of different ages with different stages of frailty [16]. Patients living in nursing homes or residential care, as well as those considered too frail (with a Rockwood [16] frailty score of 7 or higher), were excluded. Frailty was assessed by the GPs and medical assistants using a template [17] before approaching the patients about possible participation.

### 3.1 Data Collection and Analysis

After providing written informed consent, patients received two ACP facilitation appointments at their GP practices. GPs completed a brief questionnaire on diagnoses, frailty and prior ADs for recruited patients. A trained research associate (Master's in Social Sciences) conducted three in‐depth telephone interviews per participant:

◦ First telephone interview: before ACP facilitation.
◦ Second telephone interview: immediately after ACP facilitation.
◦ Third telephone interview: 12 months after ACP facilitation.

All patients meeting the inclusion criteria and with scheduled ACP facilitation in the participating practices between April 2022 and December 2022 were invited to participate in the study. We aimed for data saturation in the interviews immediately before and after the ACP conversation. However, this was not fully achievable for the 1‐year follow‐up interviews, as these took place after patient recruitment had ended. The overall number of participants was constrained by the early stage of implementation of qualified ACP in general practice, which limited the pool of available practices and patients.

Following the first interview, participants completed a short socio‐demographic questionnaire by phone. Interview guides and questionnaires were developed through literature review, interdisciplinary focus groups and ACP expert panel and patient advisory board consultation. Topics included motivations for ACP, attitudes towards medical treatment and autonomy, as well as experiences with ADs and ACP facilitation (Appendices 2–4).

The interviews were recorded, transcribed verbatim and analysed in German using MAXQDA 2020 [18]. The findings presented in this manuscript were translated into English by the authors and subsequently reviewed by a professional native English‐speaking editor. A thematic qualitative text analysis according to Kuckartz was used [19]. Initially, data were coded using deductive categories derived from the three interview guides, with coding sessions involving the expert panel to refine the system. In the subsequent iterative process, inductive categories additionally emerged from the data, resulting in 26 final categories (7 inductive and 19 deductive). In a final step, all data were coded according to the resulting coding system, yielding a category‐based analysis along the deductive and inductive topics. Details are provided in Table 1, with an excerpt of the coding scheme presented in Appendix 5. Findings are structured according to the four research questions.

**Table 1:** Research questions and coding themes.

Research questions	Main themes			
	Inductive themes	Themes before ACP facilitation	Themes after ACP facilitation	Themes 12 months after ACP facilitation
Expectations of patients before ACP facilitation				
1. Goals, wishes and motivations: What motivated patients to engage in the ACP process?	Experiences (of others) with ADs and ACP Experience (own) with ADs and ACP Personal health End of life and needing care Treatment compliance	Wishes for the AD outcome Motivation for creating an AD Association with an AD	Motivation for creating an AD Association with an AD	
2. Preconceptions: What are the views and what is the understanding of patients in general practice regarding ACP and ADs before their ACP conversations?	Experiences (of others) with ADs and ACP Experience (own) with AD and ACP Treatment compliance	Expectations regarding ACP facilitation Wishes for the AD outcome Association with an AD		
Experiences of patients after ACP facilitation (immediately after and 12 months later)				
3. Perceptions: What factors are perceived as important for a successful ACP process?	Factors for a successful ACP process Role of healthcare proxies and family in the ACP process		Experiences with ACP facilitation Results of AD/ACP facilitation ACP facilitation via video consultation	Impact of ACP facilitation Personal and religious/spiritual wishes Changes in AD documents within a year Creation of AD in the GP's practice
4. Outcomes: What benefits do patients perceive from their ACP experience in general practice?	Treatment compliance Role of healthcare proxies and family in the ACP process	Communication about treatment preferences/ACP	Results of AD/ACP facilitation Association with an AD Communication about treatment preferences/ACP	Impact of ACP facilitation Communication about treatment preferences/ACP Individual meaning of one's AD Advice for other patients Role of AD in the last year

*Note:* Data material used to answer the four research questions (regarding all three time points): 19 deductive themes from interview guides and 7 inductive themes from data material.

## 4 Results

### 4.1 Participant Characteristics

Between May 2022 and June 2024, 24 interviews were conducted with eight participants (six females and two males, 55–79 years, mean (SD) age: 63.6 (7.0) years). Interview durations ranged from 12 min to 57 min (mean = 24 min). Most participants were married (five), while one was in a relationship and two were single. None had prior ACP experience or an AD themselves. Educational backgrounds varied: one had a university degree, one a university entrance qualification, three a secondary school‐leaving certificate, two a lower secondary certificate, and one had no school‐leaving certificate.

All participants were German; three identified themselves as Protestant, four as Catholic and one as Christian. Frailty, assessed by GPs on a frailty scale (in this study from 1 = very fit to 6 = moderately frail), classified five participants as (1) very fit, one as (2) well and two as (5) mildly frail [16]. Documented health conditions included a skull fracture, non‐terminal cancer and a status post ischaemic cerebral haemorrhage.

#### 4.1.1 Goals, Wishes and Motivations: What Motivated Patients to Engage in the ACP Process?

**Making Informed Decisions and Being Safe From Unwanted Medical Treatments**

Among patients, the expectation was that the ACP process would create a valid AD supported by professionals: ‘[T]he expectation is (laughing) that I will leave with an advance directive that is effective, legally binding […] I think these are trained people who can guide me in some way, so that I can get this advance directive done’ (patient 1). Ensuring that treatment aligns with their preferences is critical: ‘I want to have EVERYTHING prepared, […] so that no one can do whatever they WANT with me. That's very important to me’ (patient 4). Similarly, not wanting to suffer is a key factor: ‘I don't want to just lie there, vegetating, with no hope of recovery’ (patient 2). Another participant articulated his wish to die peacefully without life‐support machines: ‘My wish is to peacefully fall asleep, no pain, no tubes, no machines. They should let me pass peacefully. No more injections, no artificial feeding. If there is no more hope, then people should be allowed to die’ (patient 7).

**Relieving the Burden on Healthcare Proxies and Loved Ones**

A strong motivation for creating an AD is the desire to relieve the burden on loved ones. By giving clear instructions, patients aim to spare family members from moral conflict when making medical decisions: ‘[…] I want to make sure that my husband and son don't face a moral conflict when making decisions […] by making those decisions for them in advance’ (patient 8). Patients also worry about becoming a burden to others: ‘Under no circumstances do I want to end up in a vegetative state or in a wheelchair, unable to take control of my life, whether it's needing care, being unable to care for myself, or relying on others for help’ (patient 8).

Past health crises likewise serve as a motivator to pursue an AD: ‘Because we've been putting it off for so long, even before the brain haemorrhage, […] For my family, for myself, I just want to get it done now’ (patient 8).

In summary, participants are motivated to engage in the ACP process because they expect to get a valid AD to prevent unwanted medical interventions. Personal experiences, whether related to their own health or after witnessing others' health crises, play a critical role in prompting individuals to make an AD. A key motivator is the desire to reduce the emotional burden on family members by sparing them difficult medical decisions or end‐of‐life care.

#### 4.1.2 Preconceptions: What are the Views and Understandings of Patients in General Practice Regarding ACP and ADs Before Their ACP Conversations?

**The Concept of AD Is Known, But ACP Facilitation Is New**

Participants understand ADs as being crucial for expressing treatment preferences, seeing them as tools to ensure autonomy in emergencies: ‘I imagine an advance directive as something where the doctor asks whether I have one, and THEN follows the decisions I have specified in it’ (patient 5).

However, another participant views it as a ‘dying mandate’ (patient 7), granting permission to die rather than be kept alive artificially. Regarding ACP facilitation, patients express uncertainty about what to expect. Three express concern: ‘I have no idea. I'll take it as it comes. It's scary, yeah, sure. Talking about your own death is really odd’ (patient 7). Three describe themselves as not being worried or even ‘completely relaxed’ (patient 3) or ‘unprejudiced’ (patient 5), and two participants mention that their expectation is to get information about the topic.

**Experiences With Medical Decisions With and Without an AD**

Seven participants shared past experiences of difficult decisions made for loved ones who did not have an AD at the time. These situations are emotionally challenging: ‘And WE [patient and family] would all have preferred if HE [father] had told us exactly what he wanted. Well, in writing’ (patient 2). Participants express a desire for having not only an AD when needed, but also for prior conversations about treatment preferences, as the absence of these discussions made decision‐making especially difficult. In contrast, those who had an AD for a family member feel more secure, reinforcing their decision to create one: ‘[W]hen my mother got very sick with cancer, she made an advance directive. My siblings and I all thought about it, and I decided, “I will prepare one, too”’ (patient 6).

**Obstacles for ACP: Financial Burden, Missing Guidance and Lack of Information**

While ADs provide security, creating one without guidance is overwhelming. All participants feel the need for more support: ‘[…] It's not like you can just sit down, download an advance directive from the internet, fill it out and sign it. You need to talk to a professional about what it all means exactly. I ordered one from the Federal Ministry of Justice, but it was just too complicated, so I just put it aside’ (patient 2).

One participant who had prepared an AD for her husband with a notary emphasises the importance of informed decision‐making: ‘You get a sheet of paper with all kinds of requirements on it. You sign it and that's that. […] I imagine that I can now definitely talk about and perhaps also UNDERSTAND some things’ (patient 4).

Financial costs can also be a barrier: ‘We just don't have that kind of money. Without [this study], we probably would never have made an advance directive’ (patient 7).

In conclusion, while participants value ADs, the process of creating one can be emotionally, practically and financially challenging, especially without qualified professional guidance.

#### 4.1.3 Perceptions: What Factors are Important for a Successful ACP Process?

**Conversations Guided by a Professional ACP Facilitator**

Despite initial apprehension (‘I was a bit nervous before, to be honest. I woke up in the night thinking, “Oh, what's going to happen?”’ [patient 2]), all patients feel well cared for and describe the conversations as compassionate and well‐structured, and state being satisfied with their final ADs. Facilitators' guidance helps patients reflect on their values: ‘I really liked the questioning and the doctor's way of looking at things, who questioned very precisely whether you might see it this way or that way, so that was great’ (patient 3), and ‘[I]t was exactly the right thing to do. I think it would have been very difficult without these conversations, […] to draw up this AD’ (patient 5). Time and empathy on behalf of facilitators help patients feel at ease and make for a satisfying ACP facilitation: ‘The [medical assistant/ACP facilitator] gave me time to think’ (patient 4), and ‘It was very calm. I felt very well taken care of. I was afraid, but […] she did such a great job’ (patient 6).

**Involving Healthcare Proxies in Understanding Decisions**

Involving healthcare proxies in ACP conversations ensures they understand decisions: ‘I think that's an important part for someone who is appointed as a healthcare proxy, that they know exactly how I came to this decision and why I made it’ (patient 5). Without their involvement, healthcare proxies might struggle with interpreting an AD: ‘Basically, however, I think it's better if you've spoken to the person beforehand. If only one person reads it without another person explaining “yes, that's what she meant,” it might be difficult at one point or another’ (patient 5).

However, not all patients find it easy to engage their family members in these conversations: ‘He didn't want to accompany me and I tried to talk to him a few times, but I just didn't succeed and I've now accepted that […]. I told my daughter: “There's the AD” […] but I didn't sit down and go through it with her word for word, she rejected that too, she didn't want that either’ (patient 1).

**General Practices Are the Right Place for ACP**

The general practice setting is viewed by patients as the ideal place for ACP, citing trust in their long‐term relationship with their healthcare provider: ‘[I]n the general practice there is a certain relationship of trust, so you also have the feeling that you can discuss things openly. So that was very […] helpful’ (patient 5). This sense of trust can enable patients to feel more comfortable and open during the ACP process, which is deemed essential for end‐of‐life discussions: ‘You're in good hands at the doctor's office. It's important to talk about it. You shouldn't be afraid of it’ (patient 6). One patient also emphasises the neutral framework in a GPs' practice as compared to ACP conversations at home, which helps to better reflect on and communicate wishes and thoughts: ‘[Y]ou have a bit more neutral ground [in the GP practice]. On the one hand, you have a place to talk […] yes, that's emotionally different and maybe you can think more clearly’ (patient 8).

In summary, a successful ACP process depends on several key factors: professional facilitators who provide structure, empathy and security; adequate time for reflection; a trusted, neutral environment; and involving healthcare proxies to ensure that decisions are understood and to avoid misunderstandings later on.

#### 4.1.4 Outcomes: What Benefits do Patients Perceive From Their ACP Experience in General Practice?

**A Valid AD Warranting Future Care Consistent With Care Preferences**

Patients emphasise the importance of a clear AD to ensure their wishes are respected in medical emergencies. One participant noted how the ACP process improved the quality of her AD: ‘Yes, the picture has changed insofar as the points recorded in it are now more specific. At the beginning, it was kind of a vague situation where you just said: “Okay, I don't want life‐prolonging measures.” That's a general statement. Now it's specific […] I wouldn't have known that before. If I had done it alone, I would have written something general, and I think that would have put my healthcare proxy in a difficult situation in a real case’ (patient 5). While the primary perceived benefit was an enhanced clarity of the AD, the ACP facilitation also includes guidance on its ongoing review and potential for modification: one patient reported making a minor change in her AD within the first 12 months after ACP facilitation.

**Peace of Mind**

A successful ACP process results in an AD that provides patients with a sense of security: ‘[I]t's good that it's there and if something happens to me, anyone can take a look at it [AD], […] that gives me a really strong sense of security’ (patient 2).

One participant describes the peace of mind it brought: ‘Peace of mind for myself. No more question marks. A sense of security, knowing that if something happens, I'll be in good hands, whether with my son, husband, or the [GP/ACP facilitator]’ (patient 8).

ADs are seen as a tool to settle certain aspects of one's life, perhaps comparable to a will or durable power of attorney: ‘This means that I have put things in order at this point. If I didn't have the advance directive that would be a worry for me […]’ (patient 1).

**Overcoming Barriers to ACP Communication**

Although all participants intend to discuss their wishes with loved ones, emotional barriers often delay these conversations: ‘It's always about finding excuses to finally get it started’ (patient 8). Whether immediately after ACP conversations or 1 year later, not many of the intended discussions with loved ones had taken place: ‘No, we haven't done that. It's just been daily life’ (patient 5). This indicates that ACP requires a dedicated opportunity and a safe space for discussion, as it is not considered part of ‘daily life’. One participant describes: ‘Yes, it's hard. Especially when it comes to your own death. […]. But that feeling was taken away from me because the [medical assistant/ACP facilitator] did such a great job […]’ (patient 7). This statement highlights the importance of professional ACP conversations, as such topics are rarely mentioned otherwise: ‘We don't talk about it and the good thing is that you sit in the practice and then this topic comes up because a third party is leading the conversation and I think that's good’ (patient 1). Without prior discussions, healthcare proxies or family members may have to make decisions without knowing the patient's wishes. One participant shared how family members often dismiss the topic: ‘[W]hen I say something about it, they say “Well, I'll die before you anyway, and you'll take care of everything.” So, people tend to push it aside, right?’ (patient 1). While ACP topics can be emotionally burdensome, discussing them can reduce uncertainty and anxiety: ‘It's an uncomfortable topic, otherwise more people would likely address it. […] I think once you've wrapped it up and put it in order, you can find some inner peace, instead of constantly thinking “I have to, I have to” and “what if”. It takes away some of the fear, at least for me’ (patient 8).

In conclusion, while ACP discussions are often emotionally challenging, they help alleviate fear, clarify preferences and reduce uncertainty. Professional facilitators play a crucial role in guiding patients to articulate their wishes and involve family members in the process.

## 5 Discussion

This study offers new insights into how ambulatory, elderly or frail but not terminally ill patients in primary care settings in Germany perceive ACP conversations before and after they have taken place. It highlights key factors for effective ACP facilitation, with patients expressing challenges in clearly and effectively documenting their preferences, underscoring the need for structured guidance. While ACP conversations were often perceived as emotionally challenging, they were also seen as liberating, providing a valuable opportunity to discuss wishes, values and future treatment in a trusted, professional environment. This opportunity, preferably involving important family, was highly appreciated, while talking to family members about ACP on one's own account before or after the facilitated conversations was often found difficult.

A key insight confirmed by this study is the significant barrier of initiating conversations without a facilitator, even among the motivated individuals. This underscores the critical role of professional, qualified facilitation, which participants in our study found provided essential structure, empathy and a safe space, particularly valued within the trusted general practice setting.

Our findings align with the goals of ACP outlined by the international consensus led by the European Association for Palliative Care, which defines ACP as a process for identifying values and defining future medical goals, in discussion with the patient's family and healthcare providers [6]. Recognising and supporting this processual nature of ACP is essential for developing care models that allow preferences to be clarified and adapted over time.

Therefore, to improve ACP acceptance and the creation of specific, actionable ADs, efforts should focus on embedding such facilitated support and involving family within primary care. These findings also underscore the vital role of family medicine in ACP, including facilitating family conferences [20] and actively involving healthcare proxies. Patients' continued difficulty involving family post‐ACP, despite their desire, also suggests that facilitators could proactively offer to moderate further discussions with loved ones, even after AD finalisation.

Consequently, addressing the public awareness and understanding of ACP should emphasise not just the existence of ADs, but the significant benefits of a guided process in translating general wishes into clear directives, thereby supporting both patients and, ultimately, healthcare providers in adhering to those wishes in clinical practice [21].

Research from other studies reveals a wide range of patient experiences with ACP conversations, with some avoiding discussions, others embracing them, and some initiating conversations independently [22]. While some patients report relief, security and control, others find ACP irrelevant or struggle with decision‐making when they are not in a critical situation [23]. Common reasons for avoiding ACP include perceived irrelevance, uncertainty about timing and lack of opportunity [24].

In this study, patients reported that having a concrete, personalised AD gave them a reassuring sense of control and protection. The documentation of their wishes not only alleviated anxiety about future medical situations but also reduced fears of receiving unwanted treatments. This sense of security was further strengthened by the structured nature of the facilitated conversations, which provided sufficient time for thoughtful reflection and decision‐making. Patients valued the facilitators' ability to ask nuanced questions and guide the discussion in a way that helped them feel confident and supported in formulating their preferences.

Most ACP studies focus on patients with a life‐limiting disease [25]. In comparison, the elderly and/or frail, but not severely or even terminally ill, ambulatory participants of this study proactively sought ACP primarily for future preparedness rather than for immediately foreseeable crisis management. When introduced in an ambulatory setting to patients without severe illness, ACP can be framed as a routine part of healthcare rather than an urgent response to an imminent crisis, potentially increasing engagement [26, 27].

Routine ACP should be proactively offered to chronically ill, elderly individuals and those who have experienced health crises. These groups may particularly benefit from early engagement in ACP while they still have the capacity to express their values and preferences. Importantly, ACP should not be viewed as a static event, but as a process that requires regular review and potential revision in response to changes in health status, personal circumstances or new information [6, 28]. Incorporating periodic reassessment into clinical routines may help ensure that patients' documented wishes remain current and relevant.

A key question for patients remains whether documented preferences will ensure treatment compliance when they are unable to consent. Research suggests that under certain circumstances, ACP may reduce life‐sustaining treatments, increase hospice and palliative care use, and lower hospital admissions [25, 29, 30, 31]. At the same time, evidence from a recent trial in German nursing homes and other studies [32] indicate that for individual treatment preferences laid down in ACP documents to be known and honoured by relevant medical teams, whether of emergency medical services or hospitals, systematic institutional implementation of ACP is required in addition to individual ACP conversations [33, 34, 35, 36, 37].

In summary, this study indicates that a structured, professionally guided approach to ACP in general practice, strengthened by involving family members and healthcare proxies, is well‐received and highly appreciated by patients without a terminal illness. However, the successful implementation of ACP in general practice requires appropriate training and resources, including time and financial investment.

Future research is needed to identify which patients benefit most from structured ACP interventions in general practice. This includes understanding variations in readiness, health literacy and cultural attitudes towards end‐of‐life planning. In addition, future studies should investigate the optimal timing for introducing ACP in the course of illness or ageing, and evaluate the resource implications, including time, staffing and financial investment, necessary for sustainable implementation. Such insights could guide the development of efficient, targeted models of ACP that maximise patient benefit and system‐level feasibility.

## 6 Strengths and Limitations

This study employed qualitative methods to gain insights into the perspectives of patients regarding ACP facilitation. However, the findings should be considered within the context of the study's limitations. Due to the novelty of qualified ACP facilitation in general practice, the findings are based on the experiences of eight participants located in North Rhine‐Westphalia, Germany, with three highly qualified and experienced ACP facilitators in two general practices, representing one specific ACP programme. As this did not suffice for formal data saturation, including more patients might have provided a wider range of experiences. As a result, certain perspectives or experiences may not have been captured, potentially limiting the completeness and depth of the thematic analysis. This impacts the interpretative rigour of the study, as nuanced or minority viewpoints may remain unexplored. Therefore, the conclusions should be regarded as exploratory rather than definitive. Further research with a larger and more diverse sample is recommended to strengthen the validity and transferability of the findings. Experiences with other programmes or less qualified ACP facilitators may also be different. Also, patients who are less motivated to engage in ACP facilitation may have a different outcome of the process. Furthermore, our sample was fairly homogenous in terms of the cultural background and religious beliefs shared by the participants. These variables are also known to have an impact on experiences with ACP facilitation [38].

Despite these limitations, various methods were employed to enhance the quality of the study, including a stepwise participatory approach that involved stakeholders such as ACP experts, GPs and patients, thereby ensuring that the study design, data collection and analysis processes align with the needs and perspectives of all parties involved, thus significantly improving research feasibility and relevance. This study provides rare and relevant insights into qualified ACP facilitation in German general practices, which is still infrequently provided.

## 7 Conclusion

Structured ACP facilitation is recommended in primary care settings for the elderly, especially for those with frailty and/or chronic but not terminal disease. Many patients approach ACP with unclear expectations, highlighting the need for better information about the process and its potential benefits for both patients and healthcare providers. The emotional challenges and medical complexities associated with ACP highlight the crucial role of trained facilitators. The patients of our study greatly appreciated the structured support they received, emphasising the importance of qualification for GPs and their teams or other ambulatory healthcare providers to ensure effective ACP facilitation in primary care. Furthermore, structured ACP discussions may contribute to patient‐centred care by ensuring that treatment preferences for health crises that render the individual incapable of decision‐making are respected. Patients valued the outcomes of their discussions, such as personalised and detailed ADs, underlining the need for quality standards that go beyond basic checkbox forms, which often fail to capture the complexity of individual needs. To effectively implement ACP, it is important to address both the emotional and practical challenges faced by patients and healthcare providers. Bridging the gap between documented preferences and their application in clinical practice is also crucial, warranting further research and policy initiatives to support the widespread adoption of ACP as part of regular healthcare.

## Author Contributions

A.S., K.W., Ji.dS., K.G. and A.M. contributed to the study conception, design and development of study materials. A.S. was responsible for data collection and performed the data analyses with technical support from K.W. A.S. wrote the original manuscript draft. Ji.dS. and A.M. supported the drafting of the original manuscript. All authors reviewed, critically revised and approved the final version of the manuscript for submission.

## Ethics Statement

The study was approved by the local ethics committee: Witten/Herdecke University, 7 August 2021, ref. 153/2021.

## Consent

Written informed consent was obtained from the participants before the start of the study.

## Conflicts of Interest

Kornelia Götze and Jürgen in der Schmitten are ACP trainers and received professional fees for conducting ACP qualifications, talks or workshops on ACP outside the study. Jürgen in der Schmitten (2011–2021) and Kornelia Götze (since August 2021) are treasurers of the international ACP Society (www.acp-i.org). Jürgen in der Schmitten (since February 2017) and Kornelia Götze (since June 2021) are committee members of the ACP Germany Society (www.acp-d.org). Klaus Weckbecker and Alexandra Schmidt (November 2022 to March 2023) have undergone ACP facilitator training according to the quality standards of ACP Germany. Achim Mortsiefer has no competing interests.

## Supporting information

Appendix_1_COREQ_checklist_not_anonymized.

Appendix_2_Interview_guide_before_ACP_facilitation.

Appendix_3_Interview_guide_after_ACP_facilitation.

Appendix_4_Interview_guide_12_month_after_ACP_facilitation.

Appendix_5_Quotations_illustrative_for_the_themes_and_key_questions.

## Data Availability

The authors have nothing to report.
