# Supplementary material for: Advance Care Planning in German General Practice: A Longitudinal Qualitative Study on Patients' Expectations and Experiences
Source: Health Expect. 2025 Aug 17;28(4):e70392. doi: 10.1111/hex.70392 (PMC12358674; doi:10.1111/hex.70392)
Supplement: Supplementary file 1 — Appendix_1_COREQ_checklist_not_anonymized. [file HEX-28-e70392-s002.docx]

| **No** | **Item** | **Response** |
| --- | --- | --- |
| **Domain 1: Research team and reflexivity** | | |
| *Personal Characteristics* | | |
| 1. | Interviewer/ facilitator | The interviews were conducted by AS after training and under supervision of AM. |
| 2. | Credentials | AS: Master’s degree in social science; KW: Professor, medical doctor; JidS: Professor, medical doctor; KG: medical doctor; AM: Professor, medical doctor |
| 3. | Occupation | AS is a research associate in Institute of General Practice and Primary Care (iamag); KW is the head of Chair of General Practice I and Interprofessional Care in Institute of General Practice and Primary Care (iamag); JidS is the Head of Institute of Family Medicine/General Practice; KG is a post-doc research associate in Institute of General Practice (ifam), Centre of Health and Society (chs); AM is the head of Chair of General Practice II and Patient-Centeredness in Primary Care, Institute of General Practice and Primary Care (iamag) |
| 4. | Gender | Two researchers were female, three were male. |
| 5. | Experience and training | KG and JidS are ACP facilitator, trainers and ACP trainer-trainer. JidS (2011-2021) has been and KG is (since 08/2021) treasurer of the international ACP-i society. JidS (since 02/2017) and KG (since 06/2021) are committee members of the Advance Care Planning Germany society. KW and AS (11/2022-03/2023) have undergone ACP facilitator training offered by Advance Care Planning Germany. |
| *Relationship with participants* | | |
| 6. | Relationship established | The interviewer did not have a prior relationship with the participants. The interviewer was not involved in the recruitment of patients. |
| 7. | Participant knowledge of the interviewer | Participants were aware the interviewer and other researchers were conducting a research project with the aim to understand more about the participants perspective on ACP in general practice. |
| 8. | Interviewer characteristics | The participants were aware the interviewer has no medical background and the aim of the research team was to publish this research data, which was also part of a dissertation. |
| **Domain 2: study design** | | |
| *Theoretical framework* | | |
| 9. | Methodological orientation and Theory | An inductive and deductive thematic qualitative text analysis according to Kuckartz was used. ^22^ The analysis was continuously discussed and refined with an expert panel. |
| *Participant selection* | | |
| 10. | Sampling | Participants were selected using convenience sampling. Participants of this study are patients who got an ACP facilitation in GP practices in Germany that offer ACP facilitation according to the standards of ACP Germany by trained GPs or trained medical assistants. |
| 11. | Method of approach | Participants were approached by telephone. |
| 12. | Sample size | 8 participants. |
| 13. | Non-participation | All approached patients wanted to participate. |
| *Setting* | | |
| 14. | Setting of data collection | Data was collected by telephone. |
| 15. | Presence of non-participants | During three of 24 interviews, relatives were present for a short amount of time. |
| 16. | Description of sample | 8 German General Practice patients (six female, two male, 55-79 years, mean age: 63.6 years, frailty score from 1 to 5). |
| 17. | Interview guide | We provided the interview guides (appendices 2-4). |
| *Data collection* | | |
| 18. | Repeat interviews | No repeat interviews were carried out. |
| 19. | Audio/visual recording | Interviews were audio-recorded, downloaded onto a secure folder at the University Witten/Herdecke and subsequently deleted from the recording device. The interviews were transcribed by AS. |
| 20. | Field notes | No field noted were collected. |
| 21. | Duration | Interview duration was on average 24 minutes. |
| 22. | Data saturation | Not applicable. |
| 23. | Transcripts returned | Transcripts were not returned to participants. |
| **Domain 3: analysis and findings** | | |
| *Data analysis* | | |
| 24. | Number of data coders | AS coded the data. Data was coded regularly in meetings with the ACP Expert Panel. |
| 25. | Description of the coding tree | We provided the coding tree along with the illustrative quotations (Appendix 5). |
| 26. | Derivation of themes | Themes were derived from the interview guides and the interview data. |
| 27. | Software | MAXQDA 2020 |
| 28. | Participant checking | Participants did not provide feedback on the findings. |
| *Reporting* | | |
| 29. | Quotations presented | Appendix 5 shows illustrative quotations for each theme with study numbers for participant identification. Besides, in the text short quotes are shown for additional illustration. |
| 30. | Data and findings consistent | According to the research team data presented and findings are consistent. |
| 31. | Clarity of major themes | We identified 19 deductive themes from interview guides and 7 inductive themes from data material shown in table 1. |
| 32. | Clarity of minor themes | Not applicable |
