# Supplementary material for: Advance Care Planning in German General Practice: A Longitudinal Qualitative Study on Patients' Expectations and Experiences
Source: Health Expect. 2025 Aug 17;28(4):e70392. doi: 10.1111/hex.70392 (PMC12358674; doi:10.1111/hex.70392)
Supplement: Supplementary file 2 — Appendix_2_Interview_guide_before_ACP_facilitation. [file HEX-28-e70392-s001.docx]

**Appendix 2: Interview guide: Before ACP facilitation**

**Guideline for semi-structured interview for patients who have undergone ACP facilitation within the evaACP study; Timepoint: Before ACP facilitation** (translated from German Language)

| **Research Questions** | **Guideline Questions** |
| --- | --- |
| **Motivation**  From what motivation or occasion did patients schedule the appointment for the advance directive?  **Attitude**  How do patients feel about decisions on medical treatment?  What do patients associate with patient autonomy? | First of all, please tell me what expectations and ideas you have going into the conversations in your GP practice with [Nurse/GP/ACP facilitator]?  Was there a reason why you made this appointment?  Would you have made the appointments if your GP had not approached you?  Have you already discussed your wishes and ideas regarding medical treatment with others (possibly a proxy)?  How do you feel going into the conversations with your GP?  Is there anything that is particularly important to you for these conversations? |
| **Process**  What expectations do patients have for the ACP conversations? | What expectations do you have of the conversations?  Is there someone you would like to include in the conversation? |
| **Outcome**  What outcome do the patients hope for from the ACP facilitation?  **Attitude**  What do patients associate with ACP or AD? | What would you like to regulate in your advance directive? What do you hope to achieve with the conversations?  Have you already had experience of drawing one up?  In what form would you like to document your wishes?  What would you like to discuss with [Nurse/GP/ACP facilitator]?  What do you want to have in your hands at the end? |
