# Supplementary material for: Advance Care Planning in German General Practice: A Longitudinal Qualitative Study on Patients' Expectations and Experiences
Source: Health Expect. 2025 Aug 17;28(4):e70392. doi: 10.1111/hex.70392 (PMC12358674; doi:10.1111/hex.70392)
Supplement: Supplementary file 3 — Appendix_3_Interview_guide_after_ACP_facilitation. [file HEX-28-e70392-s005.docx]

**Appendix 3: Interview guide: After ACP facilitation**

**Guideline for semi-structured interview for patients who have undergone ACP facilitation within the evaACP study; Timepoint: Immediately after ACP facilitation** (translated from German Language)

| **Research Questions** | **Guideline Questions** |
| --- | --- |
| **Process**  What experiences have patients had with ACP facilitation? | We talked last time in [time point/date]. After that, you had 2 appointments in your GP’s office.  We talked for the last time in mid-December last year. After that, you had 2 appointments in the practice  If you think back to your expectations of the previous appointments, what were they like and were they met?  Did you have someone with you at the appointments?  How did you feel at the appointments? (Room, duration, personal level)  What feelings and thoughts did you leave with? |
| **Outcome**  What outcome do patients get from ACP facilitation?  **Communication**  Have the patients communicated about topics related to ACP/AD?  **Attitude**  How do patients feel about making decisions on medical treatment?  What do patients associate with ACP or AD?  What do patients associate with patient autonomy?  What do patients associate with video consultations on ACP? | What is the most important thing you have discussed? Why is that?  Is there any content that you would have liked to discuss in more depth?  Were there any situations for which you did not want to make decisions? Why was that the case?  Are you satisfied with what you now have in your hands?  What would you describe as a advance directive after the appointments?  Have you already spoken to others (possibly proxies) about your wishes and ideas? Do you plan to do so?  To what extent do you think your advance directive will help to ensure that your treatment wishes are implemented if you are no longer able to decide for yourself?  Can you imagine having the appointments you had via video consultation? |
