# Supplementary material for: Advance Care Planning in German General Practice: A Longitudinal Qualitative Study on Patients' Expectations and Experiences
Source: Health Expect. 2025 Aug 17;28(4):e70392. doi: 10.1111/hex.70392 (PMC12358674; doi:10.1111/hex.70392)
Supplement: Supplementary file 4 — Appendix_4_Interview_guide_12_month_after_ACP_facilitation. [file HEX-28-e70392-s003.docx]

**Appendix 4: Interview guide: Twelve months after ACP facilitation**

**Guideline for semi-structured interview for patients who have undergone ACP facilitation within the evaACP study; Timepoint: Twelve months after ACP facilitation** (translated from German Language)

| **Research Questions** | **Guideline Questions** |
| --- | --- |
| **Impact**  What impact did the ACP facilitations have had on the patients in the last 12 months? | About a year ago, you had the conversation about your Advance Directive at your GP's office, which was on [date]. Perhaps you could tell me about it in retrospect. How did you feel afterwards?  Have there been any changes or experiences that have had a significant impact on you in the last year? Have there been any changes in your health?  Has the topic of “ advance directives” had an impact on you in the past year?  In the ACP conversations, you have the opportunity to talk about religious, personal or spiritual attitudes and document them. Have you also made a record of this?  Have you had any changes made to the contents of your advance directive in the past year?  Is there anything that you might want to adapt/change? |
| **Communication**  Have the patients communicated about topics related to ACP/AD in the last 12 months? | How have you talked to others about your experiences (with making an advance directive)?    Have you talked to others about what is important to you in terms of your preferences for treatment? |
| **Attitude**  How do patients feel about ACP/AD? | What does it mean to you personally to have this advance directive?    What meaning did it have for you that you had the ACP conversations on the Advance Directive at your GP practice?  If you could give other people a piece of advice from your experience with the ACP process (what was particularly valuable for you) - what would it be? |
