# Supplementary material for: Advance Care Planning in German General Practice: A Longitudinal Qualitative Study on Patients' Expectations and Experiences
Source: Health Expect. 2025 Aug 17;28(4):e70392. doi: 10.1111/hex.70392 (PMC12358674; doi:10.1111/hex.70392)
Supplement: Supplementary file 5 — Appendix_5_Quotations_illustrative_for_the_themes_and_key_questions. [file HEX-28-e70392-s004.docx]

### Appendix 5: Quotations illustrative for the themes and key questions

| **Inductive categories** | |
| --- | --- |
| Experiences with ADs and ACP (of other persons) | *“And WE [patient and family] would all have preferred if HE [father] had told us exactly what he wanted. Well, in writing, in this case. That he would have written, in this situation I want you to do this and that" (patient 2).*  *“We did it with a notary for my husband, and it was completely different. It was just a formal process, no advice was given, no time to think or share experiences. I think that’s really important.” (Patient 4)* |
| Experience with ADs and ACP (own) | *“[…] It’s not like you can just sit down, download an advance directive from the internet, fill it out, and sign it. You need to talk to a professional about what it all means exactly. I ordered one from the Federal Ministry of Justice, but it was just too complicated, so I just put it aside” (patient 2).* |
| Personal health | *“A key experience was perhaps when I was young. I once fell off one of those gymnastic bars while playing, I'll say, at school. My head hit a radiator and I fainted. What I couldn't do/ I could hear everything, but I couldn't even blink. Or make myself heard somehow. But I heard everything. And it was like this, it's such an impression for me, I'm going to say in quotes, a coma, it's also a nightmare for me somewhat.” (Patient 8)*  *“I'm not doing so well health-wise, physically, I can tell.*  *[...] And mentally, I don't know. I need to sit down in a corner, cry and let go. I'm not that far yet. It's a change, you're no longer able to cope with everything the way you used to.” (Patient 4)* |
| End of life and being in need of care | *“There are things at the end of life that you really want to do and once you've done that, it's/ at least for me, it's kind of a relief. I also think you/ Well, I have a family too, I have children and I want to/ I want to relieve them of that.” (Patient 1)*  *"Under no circumstances do I want to end up in a vegetative state or in a wheelchair, unable to take control of my life, whether it’s needing care, being unable to care for myself, or relying on others for help. I've said that’s something I don’t want. [...] I don’t want that for myself or my family." (Patient 8)*  *: "My wish is to peacefully fall asleep, no pain, no tubes, no machines. They should let me pass peacefully. No more injections, no artificial feeding. If there is no more hope, then people should be allowed to die." (Patient 7)* |
| Factors for a successful ACP process | *“We don't talk about it and the good thing is that you sit in the practice and then this topic comes up because a third party is leading the conversation and I think that's good.” (Patient 1)*  *“If I had done it alone, I would have written something general, and I think that would have put my healthcare proxy in a difficult situation in a real case." (Patient 5)*  *“The [ACP facilitator] gave me time to think.” (Patient 4)*  *“It was very calm. I felt very well taken care of. I was afraid, but […] she did such a great job.” (Patient 6)* |
| Treatment Compliance | *"It is important to me that everything is so clearly defined that there can be no confusion afterwards." (Patient 3)*  *"I want to have EVERYTHING prepared, [...] so that no one can do whatever they WANT with me. That’s very important to me" (Patient 4)* |
| Role of healthcare proxies and family in ACP process | *“In the end, it's important to me [...] that there can be no more irritation later on. [...] [F]or our son mainly, of course, right? And for each other too, of course. [...] And you can really structure that a bit, so that you have a guideline for yourself or how you can meet your partner's wishes. That's our intention behind it.” (Patient 3)*  *“So he [spouse] wasn't at this appointment. He didn't want to accompany me and I tried to talk to him a few times, but I just didn't succeed and I've now accepted that [...] I told my daughter: 'There's the AD' [...] but I didn't sit down and go through it with her word for word, she rejected that too, she didn't want that either.” (Patient 1)*  *“Because he was also involved in the process and I think that's an important part for someone who is appointed as a healthcare proxy, that they know exactly how I came to this decision and why I made it.” (Patient 5)* |
| **Themes prior to ACP facilitation** | |
| Expectations towards the ACP facilitation | *"Yeah, it's scary, of course. Talking about your own death is really a strange thing. But expectations? I'll just let it come to me." (Patient 7)*  *“So the expectations are actually that I've been working on drafting a advance directive or a power of attorney for care and have tried to find out a bit about it MYSELF [...] but haven't quite got my head around the many offers there are and the different documents and various recommendations from different institutions.” (Patient 5)* |
| Wishes for AD outcome | *“Well, I would like to make sure that if someone suddenly falls seriously ill, that you have a guideline for yourself and for the doctor, that you know that this is what we discussed, this is how we want to do it, that's the point of the whole process.” (Patient 3)*  *"Yes, well, the idea is definitely to have some control. If I can no longer make decisions myself, what, yes, what kind of guidelines I give someone so that they can make a decision, that's it." (Patient 5)*  *“So the expectation is (laughing) that I will leave with an advance directive that is effective, legally binding [...] I think these are trained people who can guide me in some way, so that I can get this advance directive done.” (Patient 1)* |
| Motivation for creating an AD | *"It's an uncomfortable topic, otherwise more people would likely address it. But it has to be dealt with eventually. We keep postponing it. I think once you've wrapped it up and put it in order, you can find some inner peace, instead of constantly thinking 'I have to, I have to' and 'what if.' It takes away some of the fear, at least for me." (Patient 8)*  *"Yes, the reason is [...] when I think of my old friend, he really lay there for half a year, and during that half year, he was completely unaware of everything. He didn't have an advance directive, and he was hooked up to tubes and machines. And it's things like that that I don't want for myself either." (Patient 7)*  *„And for me, it's just that I want to get it done. I want to put things in order. You also make a testament, or we have made a testament. And for me, that's just part of making provisions and then, in a way, taking something off my children's shoulders.” (Patient 1)* |
| Association with an AD | *“I imagine an advance directive as something where the doctor asks whether I have one, and THEN follows the decisions I have specified in it.” (Patient 5)*  *"You get a sheet of paper with all kinds of requirements on it. You sign it and that's that. There's not much explanation. You know? I imagine that I can now definitely talk about and perhaps also UNDERSTAND some things." (Patient 4).*  *“We just don’t have that kind of money. Without [this study], we probably would never have made an advance directive” (Patient 7)* |
| Communication about treatment preferences/ACP | *“And that's why it's absolutely essential that we sit down at the table together, because we can really put it on paper. [...] Now the thought has occurred to me that it's just before Christmas, so I'm not going to bring my loved ones to the table and say: “ Could we do that”. And another one of those things that you always push, or that I always look for excuses to finally get it on the way.” (Patient 8)*    *“No, we haven't. My wife and I talk about it from time to time, in general, how you imagine it. But it hasn't been anything concrete.” (Patient 3)* |
| **Themes after ACP facilitation** | |
| Experiences with ACP facilitation | *"[...] it's really weird to talk about things like that. I already have a bit of tension on my stomach. But the woman [ACP facilitator] did such a great job. It was very, very good. We discussed everything that needed to be discussed. And I'm very, very satisfied. Because then you always have a little bit/ you don't talk about death every day." (Patient 7)*  *They were very, very interesting, I have to say, especially the second appointment. Because that's when it got a little bit down to business. [...] I could have sat there for another hour. I liked it. [...] Very good. There's nothing to add. It was a very pleasant atmosphere. [...]. And then you went home heavy with thoughts and had to sort it all out again. [...] And now I have to process it again for a few weeks (laughing).” (Patient 3)* |
| Results of AD/ACP facilitation | *"So the most important thing I've kept in mind is that I would like to go to a hospice. Just like my mother. [...] Then she wrote it all down and then I also had to sign it with my brother. Yes, and then it was actually great. I left with a really great feeling." (Patient 6)*  *"The most important thing is that I've done something in case I do end up in hospital and I can then decide for myself that there's someone there to look after me. [...] Yes, it was really important to me. It's important to me because I've been through it all and it's not funny. When you have NOTHING in your hands." (Patient 4)*  *“Basically, however, I think it's better if you've spoken to the person beforehand. If only one person reads it without another person explaining “yes, that's what she meant”, it might be difficult at one point or another.” (Patient 5)* |
| Motivation for creating an AD | *"[...] [T]hat I am released if I need long-term care, whatever happens, whether it's in a wheelchair or really in a nursing bed at home or a nursing home or something else. So of course I wouldn't want that for myself either. That's the whole package, I'd say that's very important for me and for my family." (Patient 8)* |
| Association with an AD | *"I would call it protection, at least ad hoc. For my healthcare decisions in the hospital." (Patient 8)*  *"[T]hat I now feel emotionally better taken care of, than with a completely different advance directive, with a notary or somewhere else. [...]. Here, you are now well informed, and that's good." (Patient 4)*  *"[T]he good thing is that it’s actually so brief, to the point, and clearly formulated, or at least visible on those sheets there. […] And I’m aware that if a situation really does arise where the advance directive is consulted, then they will know exactly what I want." (Patient 2)* |
| Communication about treatment preferences/ACP | *"Yes and no, they know I was there, I even have one [daughter] sitting next to me right now, so they all know about it, but I think we all need to sit down together and calmly talk about it. And right now, each of them has something else going on, so there isn’t the calm we need for that." (Patient 4)*  *"I haven’t. I haven’t spoken to anyone else about it either. [...] Yes, it’s been like we’ve had an incredibly busy week. [...] [A]nd I thought to myself, 'Well, this is not the time to bring up that topic,' but we’ve now booked a few days of vacation, and I really plan to bring it up with my husband calmly and ask him again how he feels about it. But at the moment, I am/ He’s also blocking it a little bit. So." (Patient 1)* |
| ACP facilitation via Video consultation | *"Oh no, I’d really like to sit face-to-face with the person and see them. [...] I find it too impersonal, although it’s nice and practical that everything is available now, I don’t think I’d be able to handle it anymore." (Patient 3)*  *"Well, I wouldn’t know the doctor, or I wouldn’t know them [ACP facilitator], right? [...] I/ Like I said, I found it extremely comfortable because I’ve known [GP/ACP facilitator] for such a long time. And he really [...] gives such a secure, good feeling. [...] But it’s possible, of course." (Patient 2)* |
| **Themes twelve months after ACP facilitation** | |
| Impact of ACP facilitation | *"In a way, it gives a bit of peace, that you’ve somewhat clarified things, though I still haven’t done it yet—I haven’t had the kids sign it. It’s still lying there in the corner. But they know where it is, and they can take it if something happens. It gives a certain peace, BUT there are also thoughts: ‘Is everything you’re doing really right?’ Well, okay, but you can always change it if you want. As long as you can, right?" (Patient 4)*  *"Well, it was very good. It was very helpful too. I was in a situation where I had only vaguely dealt with it, and through this conversation, I engaged with it much more deeply, and for me, it was a very, very good support to then make the appropriate decisions." (Patient 5)* |
| Role of AD in the last year | *“I: Has the topic of advance directives played a role for you in the past year?*  *No, no, fortunately not, no. Neither in my family nor for myself directly, no, no, no.” (Patient 1)* |
| Individual meaning of one’s AD | *"A very reassuring feeling. And I hope that […] the doctors and nurses, if it ever comes to that, and also my family, will respect my wishes and everything that I want. That is my wish." (Patient 6)*  *“I think it's good that it's there and that if something happens to me, anyone can take a look at it [AD], doctors or my husband or my son or my brother, whoever, and that it says what I would like. And that gives me a really good, secure feeling.” (Patient 2)*  *“This means that I have put things in order on this point. If I didn't have the advance directive, it would be a burden for me because I think it's important and that's one of the things I want to get done. There are things that I haven't done, such as decluttering my house.” (Patient 1)* |
| Personal and religious/spiritual wishes | *"As far as I know, no. No, nothing special.” (Patient 3)*  *"Mhm. (negating) I think I wrote that I want a/ no, I would have to read it again to see if a pastor is allowed to come. I don’t remember if it was written like that. […] But it would be nice." (Patient 4)*  *"No, not really. A religious or spiritual situation didn’t play a role in it [ACP facilitation]." (Patient 5)* |
| Communication about treatment preferences/ACP | *"We take a look at it sometimes; I have the folder here with mine in it, and then we’ll talk about what we want. Yes, yes, we do that. I’m also glad that something like this exists. Honestly, just imagine, you’re lying in the hospital, can’t talk anymore, can’t speak, and they do whatever they want with you." (Patient 6)*  *Interviewer: "So, your son knows that it exists and where it is, but you haven’t talked in more detail about the contents?"  Patient: "Not specifically, but he already knew how we imagine it. But he also works in the medical field, so it’s not unfamiliar to him. But we haven’t talked with him about it CONREATLY yet. We’ll definitely do that sometime soon or eventually." (Patient 2)*  *“So he [spouse] wasn't at this appointment. He didn't want to accompany me and I tried to talk to him a few times, but I just didn't succeed and I've now accepted that [...] I told my daughter: 'There's the AD' [...] but I didn't sit down and go through it with her word for word, she rejected that too, she didn't want that either.” (Patient 1)* |
| Changes in ADs within a year | *"In fact, my son J was missing from mine and he was in my husband's [AD]. I can't remember in what context and we had that changed." (Patient 2)*  *"No, we haven't changed anything now. [...] Well, I could imagine that if you're really confronted with an illness, you'll deal with the situation again." (Patient 5)* |
| Creation of AD in the GP’s Practice | *“[S]o it was quite natural for us to use our GP as a place to go and take care of things there. [...] [H]e knows our medical history, we don't have any major illnesses. That was a comfortable setting for us." (Patient 3)*  *“[…] It’s not like you can just sit down, download an advance directive from the internet, fill it out, and sign it. You need to talk to a professional about what it all means exactly. I ordered one from the Federal Ministry of Justice, but it was just too complicated, so I just put it aside.” (Patient 2)*  *"The [Nurse/ACP facilitator] was really very objective, also very compassionate, very patient. So that was the best place for me. It really suited me. [...] I have confidence in my GP, yes, that's why I think that's the right place. I couldn't imagine doing it with a lawyer [...]." (Patient 1)* |
| Advice for other patients | *“What I would say to them is, do the same as me, at the doctor's office, you're in good hands there. It's important to talk about it. You shouldn't be afraid of it.” (Patient 6)*  *"So first of all, I would really recommend doing it through a GP, together with them, if you have a relationship of trust with them. And then I would really just say overall, it's important to have such an advance directive so that you don't get into any situations when you're very ill that you don't want to get into." (Patient 5)* |

Data material used to answer the four research questions (regarding all three time points): 19 deductive themes from interview guides, 7 inductive themes from data material
